# The Seasonal Dynamics of Artificial Nest Predation Rates along Edges in a Mosaic Managed Reedbed

**DOI:** 10.1371/journal.pone.0140247

**Published:** 2015-10-08

**Authors:** Iain Malzer, Barbara Helm

**Affiliations:** Institute of Biodiversity, Animal Health and Comparative Medicine, University of Glasgow, Glasgow, Scotland; Hungarian Academy of Sciences, HUNGARY

## Abstract

Boundaries between different habitats can be responsible for changes in species interactions, including modified rates of encounter between predators and prey. Such ‘edge effects’ have been reported in nesting birds, where nest predation rates can be increased at habitat edges. The literature concerning edge effects on nest predation rates reveals a wide variation in results, even within single habitats, suggesting edge effects are not fixed, but dynamic throughout space and time. This study demonstrates the importance of considering dynamic mechanisms underlying edge effects and their relevance when undertaking habitat management. In reedbed habitats, management in the form of mosaic winter reed cutting can create extensive edges which change rapidly with reed regrowth during spring. We investigate the seasonal dynamics of reedbed edges using an artificial nest experiment based on the breeding biology of a reedbed specialist. We first demonstrate that nest predation decreases with increasing distance from the edge of cut reed blocks, suggesting edge effects have a pivotal role in this system. Using repeats throughout the breeding season we then confirm that nest predation rates are temporally dynamic and decline with the regrowth of reed. However, effects of edges on nest predation were consistent throughout the season. These results are of practical importance when considering appropriate habitat management, suggesting that reed cutting may heighten nest predation, especially before new growth matures. They also contribute directly to an overall understanding of the dynamic processes underlying edge effects and their potential role as drivers of time-dependent habitat use.

## Introduction

“Edge effects” are changes in ecological patterns that occur along the boundaries between different habitat types. They have been widely studied in a number of different habitats [1–4] and several kinds of edge effects have been described [5]. These may be abiotic processes, such as changes in microclimate with increasing distance from the habitat boundary [6], which in turn can have a direct consequence on the abundance and distribution of biological organisms along habitat edges [5,7,8]. Finally, changes in abundance or distribution of a given species along a habitat boundary may have indirect consequences for other organisms through an intensification or reduction in the chance of interactions between these species [5,9].

Edge effects affecting species interactions have received considerable attention [9]. For example, Fagan et al. [9] suggest several mechanisms by which species interactions may be intensified along habitat edges. Organisms may show preferences for edges if they can exploit resources from both sides of the edge, encouraging them to spend more time at edges than in interior habitats [10]. Similarly, when edges act as barriers animals often move parallel to them, treating them as ‘travel lanes’ [9], which could result in increased interspecific contacts. ‘Spillover’ edge effects can further increase opportunities for species interactions. These occur where a species common in one environment moves to a neighbouring habitat where they interact with the species’ resident in these areas [11]. Such interactions will occur most frequently at the boundary becoming more dilute towards the interior.

An example of changing species interactions along habitat boundaries are avian nest predation rates, where proximity to an edge increases the probability of a nest being predated [12]. Increased predator activity along edges, and the spillover of predators into neighbouring habitats could provide mechanisms by which encounter rates between predators and nests are increased [10]. As nest success is explicitly related to an individual’s fitness, and is thus a vital demographic rate in bird populations [13], edge effects on nest predation rates have been extensively explored. Many studies in forest and wetland habitats have found significant edge effects on nest predation [1,12,14], however, these results are far from unanimous [15,16]. It is therefore clear that the mechanisms underlying edge effects on nest predation are difficult to generalise between habitats, and that these processes are not fixed functions of edges but likely to vary both throughout space and over time [1,8,17].

To gain a comprehensive understanding of the mechanisms underlying edge effects on nest predation rates, studies should consider, and where possible incorporate a means to test, the dynamic aspects of edge effects over time and space. This may be especially important for temporal effects which have received less attention than spatial variation. For example, changes in the abruptness and extent of edges over time may affect the tendency of animals to use them as travel lanes. Or, seasonally changing dispersive behaviours of predators may mediate movements into neighbouring habitats during spillover edge effects. This study seeks to demonstrate the significance of dynamic processes underlying edge effects on nest predation rates and emphasise the importance of the incorporation of temporal variation when defining an edge effect. We look to demonstrate these effects in managed reedbeds with the additional aim that results will contribute to conservation and management decisions at these important sites.

Reedbeds are a globally threatened habitat [18] on which many specialist species rely [19,20]. This includes several bird species which breed almost exclusively in these habitats [21–23]. Consequentially, edge effects have been well studied in reedbed habitats. Batáry & Báldi [12] review studies in marsh habitats and demonstrate, overall, a significant edge effect on nest predation rates. However, subsequent experiments involving artificial nests have shown more variable results, suggesting effects may be dynamic [10,23–27]. Indeed, several studies have alluded to dynamic spatial and temporal effects within reedbeds. Báldi and Batáry [28] show that edge effects on predation rates vary between different sites. Further spatial variation may be driven by the sharpness of the edge [29,30]. Seasonal [25,30,31], and longer term [8] temporal variation have also been noted.

In reedbed habitats, edges often result from human influence, being harvested commercially or managed for long term preservation [23,32–34]. Reed cutting can provide a resistance against the natural succession of the reedbed [32]. It may also benefit breeding birds by increasing heterogeneity in the reedbed, providing birds with old reed in which to nest, and more open foraging sites [32]. However, there are potential negatives [35], with some literature suggesting a reduced arthropod and bird abundance after cutting [22,33,36]. Recently, reedbed cutting practices have been undertaken in a mosaic pattern, with only small unconnected patches of the reedbed being cut. This practice will hinder succession, while avoiding the implications for wildlife of cutting on a large scale [23].

These mosaic managed reedbeds offer a useful system in which to study dynamic edge effects. Visually, reedbeds are uniform in structure, being composed mainly of uninterrupted *Phragmites* stems. Such stands can cover vast areas with this apparently homogeneous habitat with the consequence that edges within reedbeds, such as those caused by reed cutting, are especially apparent. Further, the effects of these cut edges within reedbeds will be extremely temporal. Most commonly, dead reed stems are cut in the winter months, generating open patches prior to the growing season, which start to be filled in by reed growth beginning in mid April. By June mature new reed will have reoccupied patches cut the previous winter [32,37]. Thus, in this system, the severity of edges caused by reed cutting is expected to vary dramatically throughout the breeding season of reedbed specialist Passerines, and currently, the implications of this for their breeding biology are not well established. Further study of predation rates in reedbeds can thereby contribute to understanding dynamic edge effects in general, but is also of considerable conservation value.

The Tay Reedbeds in Scotland are cut in a mosaic pattern to promote the breeding success of the most important population of bearded reedlings, *Panurus biarmicus*, in the British Isles [38]. This specially protected species is highly localised to reedbed habitats [39,40] and may be especially susceptible to the effects of dynamic edges in mosaic managed reedbeds. It begins breeding in early April, having several broods until August [41] and will therefore be nesting during every stage of new reed growth. In April, before new growth, nests are positioned in patches of dead reed stem, often close to habitat boundaries [21] such as those between cut and uncut patches. If predation rates are mediated by edge effects along these boundaries, then the temporal changes in the structure of the reedbed over the season, should drive a dynamic, declining edge effect.

We use an artificial nest experiment conducted at the Tay Reedbeds to investigate this potentially dynamic edge effect caused by mosaic reedbed management. Artificial nest experiments have been useful when highlighting patterns in nest predation rates throughout different habitats [14,30]. They may also be especially important in reedbed habitats when access to real nests can cause considerable disturbance to nesting habitat. However, they can be misleading and subject to bias [42–44]. To mitigate these problems, this study followed the guidelines proposed by Major & Kendal [42]. At no point are quantitative comparisons drawn between predation rates of artificial nests and real nests. Also, steps are taken to avoid induced predation of artificial nests [45–47]. Finally, the experiments were based explicitly on the bearded reedling, this included imitating the design of the nest, the egg structure, the laying or placement dates, the number of broods and the incubation period.

To demonstrate this dynamic edge effect we test three hypotheses; **i)** that there is an edge effect on predation along the cut and uncut boundaries between patches of mosaic cut reedbed as measured by increasing nest predation rates closer to these edges. **ii)** That nest predation rates change over the season in association with seasonally changing characteristics of the reedbed habitat. And finally, **iii)** that edge effects on predation rates along cut and uncut boundaries are dynamic throughout the breeding season, being more pronounced early in the season, when edges are most apparent, than later in the season when reed growth is mature. This is tested by observing whether any edge effect is dependent on the level of new growth over the season. In addition to the investigation of dynamic edges in reed habitats, we also aim to assist practical reedbed conservation and management by identifying the more static characteristics of the reed that influence predation rates, such as reed density or height. This study aims to contribute to a general understanding of the mechanisms underlying dynamic edge effects, while providing relevant insight for the practical conservation of reedbed Passerines.

## Materials and Methods

### Study Site

The study took place during April-June, 2013 and 2014, in the Tay Reedbeds on the northern side of the Tay Estuary, Scotland (56°23.00', -003°10.00'). All permissions of access were granted by private land owners. The research was conducted in a protected habitat during the breeding season of several specially protected bird species. All appropriate Schedule 1 licences were granted by the British Trust for Ornithology and held by field researchers for the duration of the study. At around 4.1km^2^ these are the largest *Phragmites* reedbeds on the British Isles. During January to March of each year in the study, reed blocks were cut using a Saiga reed harvester. The cut blocks were relatively uniform in size at around 350m^2^. They were cut in a mosaic pattern with 50–100m of uncut reed between blocks and a minimum of 20m boundaries at the land and water edges. The same blocks were cut in each year apart from three larger blocks towards the west of the reedbed which were only cut in 2013.

### Artificial Nest Design

The artificial nests were modelled on a small collection of bearded reedling nests from the Tay Reedbeds and Leighton Moss. A bowl shaped base made of 25x25mm chicken wire provided structure for the nests. Small *Phragmites* stems were woven through the wires to form the main structure. Larger stems and *Phragmites* leaves were positioned at the furthest edges. The cup depression in the centre of the nest, in which the eggs would be placed, was lined with *Phragmites* panicles, it had a circumference of 5cm and was around 6cm deep (see S1 Fig).

Four eggs were placed in each nest. Three of the eggs were made of white, oven drying, clay. Bearded reedling eggs are white with small black markings. We left the eggs fully white, as the benefits of adding these small markings are likely to be outweighed by the biases to predation incurred from unnatural paint scents [46]. The clay eggs were around 20mm from the apex to the base and had a 14mm circumference at their thickest width [48]. The eggs were slightly heavier than the weights expected for bearded reedling eggs (2.5g, 1.9g, respectively). If predated, clay eggs should show imprints of teeth, claw or beak marks, allowing some insight into the extent of avian or mammalian predation [49]. A real quail’s egg was also added to each nest to provide insight into the size of the predator, where nests with predated clay eggs, but intact quails eggs, would suggest small predators [50]. In addition, nest cameras were positioned on 8 (5%) randomly chosen nests throughout the study, to record predation events and aid predator identification.

A nest was considered predated if any of the eggs were removed or there were obvious signs of predator interest such as marks or cuts on the clay eggs or the breaking of the quail eggs. After the experimental duration, if a nest was still intact it was considered not predated. Nests, eggs and cameras were left outside in a sheltered location for at least 5 days prior to their experimental placement to allow adequate ‘airing’ of the equipment before use [51]. Initial nest placement was conducted at dusk. This meant that any potential visual predators would have minimal time to relocate the nests before darkness if they should associate researchers with a food source. Subsequent nest checks were not limited to the evening, they occurred randomly during different periods of the day without any routine. In addition, rubber boots were worn and eggs were handled with gloves to minimise human scents.

### Temporal Design

Replicates of the experiment were conducted in April and June in 2013, and April, May and June in 2014. The experiment was not conducted in May 2013 due to logistical constraints. We timed the placement of the nests in April with evidence that the birds were in breeding condition from bird ringing studies. We repeated the experiment in May to correspond with estimated laying times of late first or early second broods. We then repeated the experiment in June when the majority of second clutches are expected to have been laid. All nests within each month were placed on the same day and left for 12 days, which is the typical incubation period for the bearded reedling [41]. Between years, the dates of placement within each month were similar, to within 3 days. The design of the artificial nests was kept constant between each month and year. In one month, 16 nests were placed along the edges of two different cut blocks. This meant each experimental replicate consisted of 32 artificial nests placed at two distinct sites. Subsequent analysis suggested little variation in predation patterns between spatial replicates, and so in further analysis data were pooled between sites. Nests were checked every second day, meaning an artificial nest surviving for the full 12 days, would be checked 6 times.

The study therefore has three temporal axes. i) The year, either 2013 or 2014, of the experimental repeat. ii) The month of the repeat within a given year, measured as the number of days in the given year since April 1^st^ and hereafter referred to as “April days”. Finally, iii) the number of days during a given experimental repeat that the nest had been exposed for, hereafter “exposure days”. On completion of all the experimental replicates over the two breeding seasons the fates of 160 artificial nests were available.

### Spatial Design

In each year, the experiment was conducted simultaneously at the edges of two cut blocks. Due to slightly fewer locations being cut in 2014, we used different cut blocks in 2013 to those used in 2014. In 2013 cut blocks used in each experimental replicate were 1km apart, while in 2014 they were separated by 500m. All the blocks used were similar in size and had been cut in the previous winter using the same reed harvesting machine and technique. All sites had been cut yearly following this protocol for at least five years previous to the experiment.

**Fig 1:**
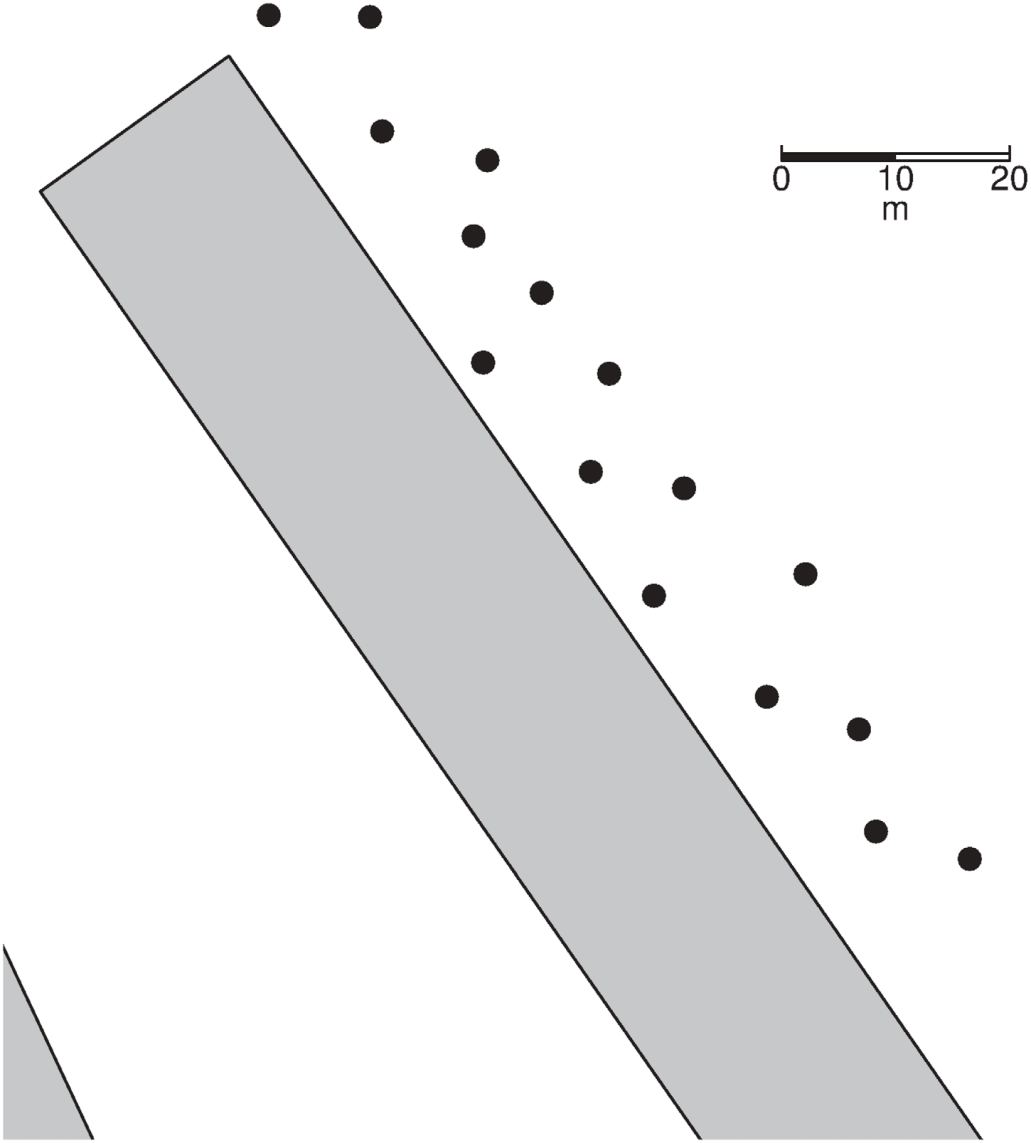
Spatial layout of artificial nests along the boundary of a block of cut reeds. The dots represent artificial nest locations, the grey rectangles are cut patches of reed, remaining, unshaded areas are unmanaged reed. Example shown is for half the nests placed in April 2013.

The artificial nests were placed in old uncut reed at differing distances from the edges of the cut boundaries up to a distance of 14 metres. This distance threshold corresponds the distances of several real nests located previously on the Tay (Malzer, personal observations). Other real nests have been located considerably deeper into the reeds, but due to the dense impenetrability of the uncut areas, any edge effects are likely to sharply decline with distance, and be captured within this distance threshold. 16 nests were placed between 0.0 and 7.0 metres from the edge, and 16 between 7.1 and 14.0 metres to ensure a uniform distribution of nests throughout the distance threshold. A random number between these boundaries generated in R 3.1.1 [52] defined the distance of the given nest. Nests were placed every 10 metres along the cut boundary at a height of 30cm off of the ground. Placement of the nests along the edge of the cut area occurred in an alternating manner with one nest being placed between 0–7 metres, and the next between 7–14 metres. This ensured there were no clusters of nests at similar depths into the uncut reed when moving along the boundaries (See Fig 1).

### Quantifying Habitat Characteristics

**Fig 2:**
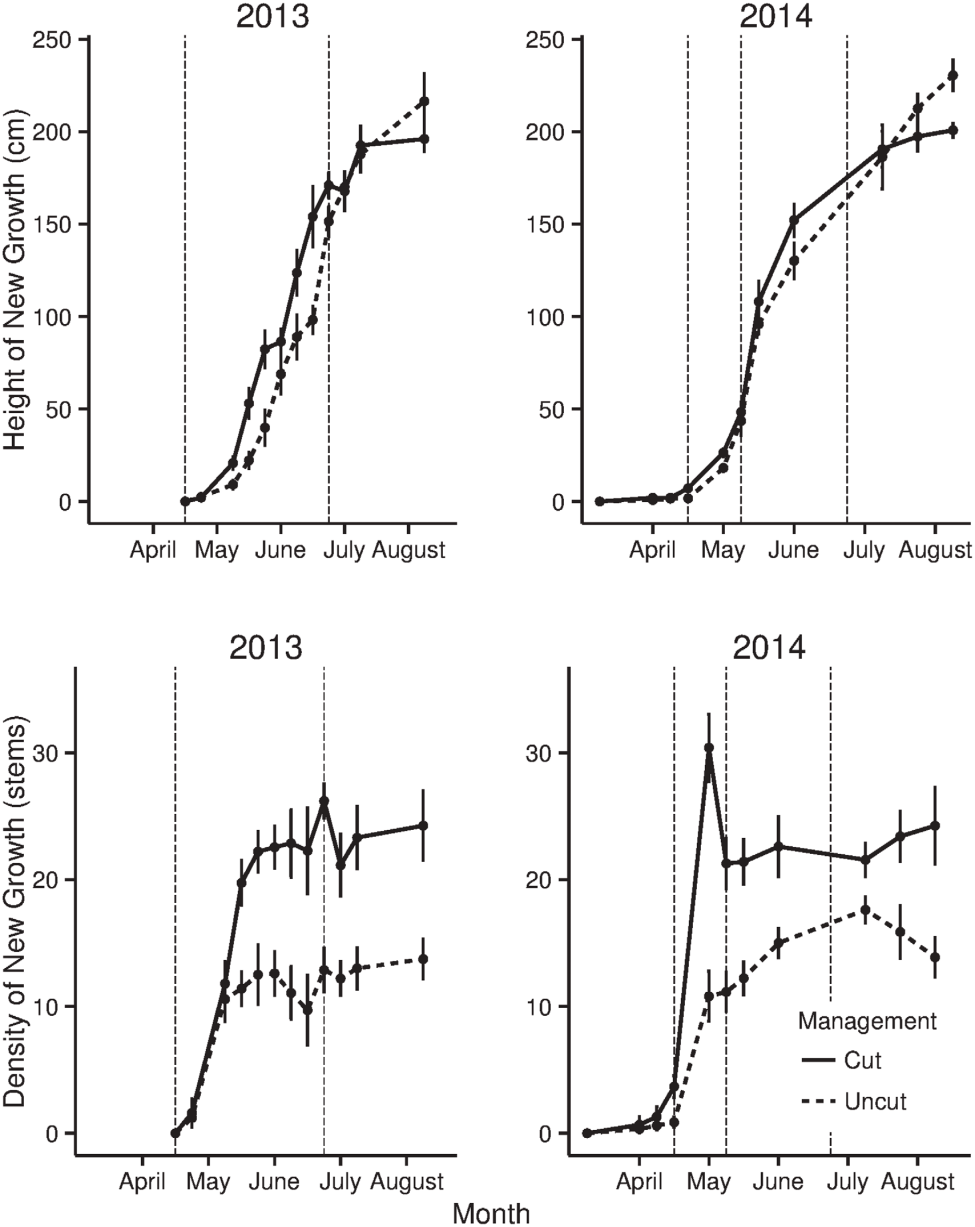
Growth of new reeds. **A** Average height and **B** density of new reed stems in each recorded week of the study in 2013 and 2014. Measurements were made on the same day for sites cut the preceding winter, and uncut reed. Vertical dotted lines represent the weeks in which the artificial nest experiments were initiated. Error bars are one standard error from the mean.

Two distinct habitat characteristic datasets were collected during the study. Data were first collected from random patches of cut and uncut reed almost weekly throughout both seasons. This allowed the growth of the reed to be quantified (Fig 2) and highlighted any differences between new growth in cut and uncut patches. Additionally, habitat measurements were also made around the artificial nest sites after the nest became inactive. The data collected around the nest sites were used to quantify reed characteristics at each nest and used in subsequent analysis to model predation rates.

The reed characteristics were measured at each random point or nest using the following methods. The heights (in cm) of five randomly selected reeds within 1m of the point were measured to obtain the average heights around the given nest. Density was measured by inserting a 1m long stick into the reeds at a height of 1m and counting the number of stems touching the stick [24]. Again five measurements per point were taken to obtain average densities. These measures were conducted for both dead and alive stems. The sum of the density of dead and alive stems gave the overall density. The depth of the litter layer was measured in three locations around the point to the nearest 5cm. This was conducted by parting the litter until the mud below was visible and measuring the depth between the upper litter and ground below [53]. Average water cover was estimated to the nearest 10% using a 0.5 x 0.5m quadrat in three locations within 3m of the artificial nest [53]. Finally, a single number between 1 and 5 was used to categorise the degree of reed stagger around each nest. As the reeds age and areas become dense with multiple years of new growth, they often bend and, in some cases areas, can become quite flattened. Areas with straight, erect reeds were given a stagger rating of 1, while the flattest areas were classed as 5 [54].

### Statistical Analysis

The differences in habitat variables collected at artificial nest sites over the given season were investigated using linear models with April days as the predictor variable. Changes between the two years of the study were investigated using Mann-Whitney tests for leaf litter and water cover, which did not meet assumptions of normality, and using T-Tests for all other variables. When comparing the differences in new reed height and density between years, only reed measurements collected during April and June were analysed. Since these specific characteristics change so extensively through the season, including the May data, collected only in 2014, would invalidate the comparison. Finally, T-Tests were used to compare height and density of mature growth collected from different areas of the reedbed between managed and unmanaged sites.

To test whether there was a temporal effect on the survival probabilities of the artificial nests driven by the month in which the nest was active, we calculated Kaplan-Meier survival functions for nests in each month [55]. This is a simple measure of the proportion of nests surviving at each nest check over the duration of their exposure. We used month as a three level categorical variable, rather than April days, as these functions cannot accommodate continuous variables. We used the nonparametric log-rank test to formally compare the survival distributions of the different levels of this covariate [56]. This gave an indication of overall temporal effects on survival within a season.

We then used Cox’s proportional hazards (CPH) models in the R package survival [57], to investigate the effects of a wider set of covariates on the tendency of an artificial nest to be predated. These semi-parametric models allow the probability per unit of time that an event will occur to be modelled as a function of a baseline hazard and a combination of either continuous variables, such as the distance from the edge of a cut area, or categorical variables, for example the year of nest placement. These survival analysis techniques have seen increasing use in nest predation studies in which the age of the nest at first encounter, and the age at failure are known [58–61], with the major motive for their use being the well developed framework and readily interpretable output [62,63].

Several of the habitat variables, namely those concerning the characteristics of the reed, were correlated and therefore could not be included as separate covariates in the CPH models. We therefore used a principal component analysis (PCA) to reduce the dimensionality of the reed characteristic covariates [64]. Covariates included in the PCA were old height, new height, old density, new density and degree of stagger. These were standardised to have a mean of 0 and standard deviation of 1 before the PCA was carried out on the correlation matrix. The importance of each resulting axis was then assessed using a parallel analysis approach (R –Package paran, [65]) where eigenvalues from the PCA were contrasted to those of 10000 simulations of normal, uncorrelated datasets with the same structure as the original data [66]. Scores from the axes deemed most important by the parallel analysis were then retained and used as continuous explanatory variables in the models.

A full CPH model was then fit including all the covariates in question, except for date which was correlated with reed regrowth. These were; the reed characteristics represented by the relevant principal component scores, water cover, leaf litter depth, distance of the nest from the edge of a cut area and the year of the study. We also included an interaction term between the principal component representing new growth (see results) and the distance from the edge of the cut area to investigate any changes in edge effects over the growing season. Adherence to the model assumption that the effect of a predictor is proportional over time, was assessed using Schoenfeld residuals [67].

Starting with the full model, we used a backwards stepwise approach based on the AIC values of each candidate model to find the most appropriate models. This was conducted using the stepAIC command in R package MASS [68].

## Results

### Reed Growth and Phenology

Between April, May and June there was a rapid increase in the biomass of reed as new growth occurred (Fig 2). New growth led to an increase in the height (*Linear Model; F*_*1*, *158*_ *= 256*.*9*, *P<0*.*001)* and total density (*F*_*1*, *158*_ *= 57*.*4*, *P<0*.*001*) of new reeds over the season. Table 1 shows the correlations between all the recorded covariates. The density and height of new reed growth showed a strong positive correlation with April days. The correlation between new height and new density was captured in the PCA. On completion of the PCA the parallel analysis for dimension reduction suggested the retention of the first three principal components. These accounted for 81% of the variation between the covariates concerning reed characteristics (Table 2). Principal component 1 (PC1) represented the new growth of reed, with low scores representing no new growth and the highest scores representing dense, high new growth. PC2 represented old reed density and the degree of stagger. PC3 represented old height only. Of the other habitat variables, water cover fell over the season at nest sites *(F*_*1*, *158*_ *= 5*.*68*, *P = 0*.*01*), and leaf litter increased (*Leaf Litter F*_*1*, *158*_ *= 4*.*96*, *P = 0*.*026)*. There was a slight decrease in the height of the old growth (*F*_*1*, *158*_ *= 3*.*61*, *P = 0*.*056*). The degree of stagger remained constant over the season (*Stagger; F*_*1*, *158*_ *= 0*.*647*, *P = 0*.*52*).

**Table 1:** Correlations between habitat covariates. Numbers represent Pearson’s r correlation coefficients. Significant correlations (P<0.01) are marked with an asterisk.

	Old Height	New Height	Old Density	New Density	Total Density	Water Cover	Leaf Litter	Degree stagger	Distance	April days
Old Height	1									
New Height	-0.21*	1								
Old Density	0.13	-0.19	1							
New Density	-0.22*	0.81*	-0.17	1						
Total Density	-0.05	0.44*	0.61*	0.60*	1					
Water Cover	0.19	-0.19	-0.02	-0.21*	-0.17	1				
Leaf Litter	-0.02	0.22	0.12	0.08	0.16	0.05	1			
Degree Stagger	0.06*	0.05	0.18	0.02	0.16	0.03	0.25	1		
Distance	0	0.14	0.16	-0.02	0.11	0.02	0.28*	0.19	1	
April Days	-0.15	0.89*	-0.08	0.79*	0.51*	-0.18	0.17	0.08	0.07	1

**Table 2:** Factor loadings of the first three principal components included in the further analysis.

Reed Characteristic	PC1 (0.40)^a^	PC2 (0.23)	PC3 (0.17)
Height of Old Reed	-0.324	0.224	0.916
Height of New Reed	0.639	0.198	0.175
Density of Old Reed	-0.280	0.558	0.289
Density of New Reed	0.637	0.189	0.157
Degree of Stagger	-0.023	0.750	0.145

^a^ Numbers in brackets are the proportion of variation in the dataset that each principal component explains.

Fig 2 shows the differences in reed growth between areas of reed that were cut the preceding winter, and uncut reed. New growth in cut areas reached a lower maximum height (*cut sites*: *217*.*16*, *uncut sites*: *241*.*25 cm*, *T-Test*_*1*, *137*_ *= -5*.*37*, *P<0*.*001)*, and higher density (*cut sites*: *27*.*6 uncut sites*: *22*.*2stems*, *T-Test*_*1*, *152*_ *= -6*.*95*, *P<0*.*001)* when mature than in uncut areas. The growth curves show that the final artificial nest experiments in June were conducted when reed was at two thirds of its maximum height.

There were differences in the phenology of new reed growth between the years of the study. In 2013 new growth appeared suddenly in mid April and rapidly increased in biomass over the growing period (Fig 2). New growth appeared around five weeks earlier in 2014, but remained at low height and density until mid April when rapid growth occurred. This meant that, despite new shoots appearing far earlier in 2014, the major growth periods were similar between the years. Of the habitat covariates collected at the sites of artificial nests, only the depth of the leaf litter differed between the years of the study (Table 3).

**Table 3:** Differences in habitat variables collected around artificial nest sites during April and June, between years of the study. P-values were calculated using T-Tests for normally distributed variables. The final two variables, in italics, did not conform to normality and so P-values are based on Mann-Whitney U tests, with W values as the test statistics.

Covariate	2013	2014	T / W Value	P-Value^a^ ^,^ ^b^
Old reed height (cm)	223.37	228.05	-0.817	0.415
New reed height (cm)	61.44	82.4	-1.099	0.27
Old reed density (no. stems)	18.52	19.57	-1.452	0.148
New reed density (no. stems)	5.16	4.3	1.105	0.271
Total reed density (no. stems)	23.6	23.8	-0.174	0.862
Degree of stagger	2.18	2.39	-0.947	0.344
*Water Cover (% of quadrat)*	*14.5*	*18.2*	*2657.5*	*0.137*
*Leaf Litter (cm)*	*11.48*	*17.0*	*2171*	*0.001*

^**a**^ P-values were calculated using T-Tests for normally distributed variables.

^b^ The final two variables, in italics, did not conform to normality and so P-values are based on Mann-Whitney U tests, with W values as the test statistics.

### Nest Predation

**Fig 3:**
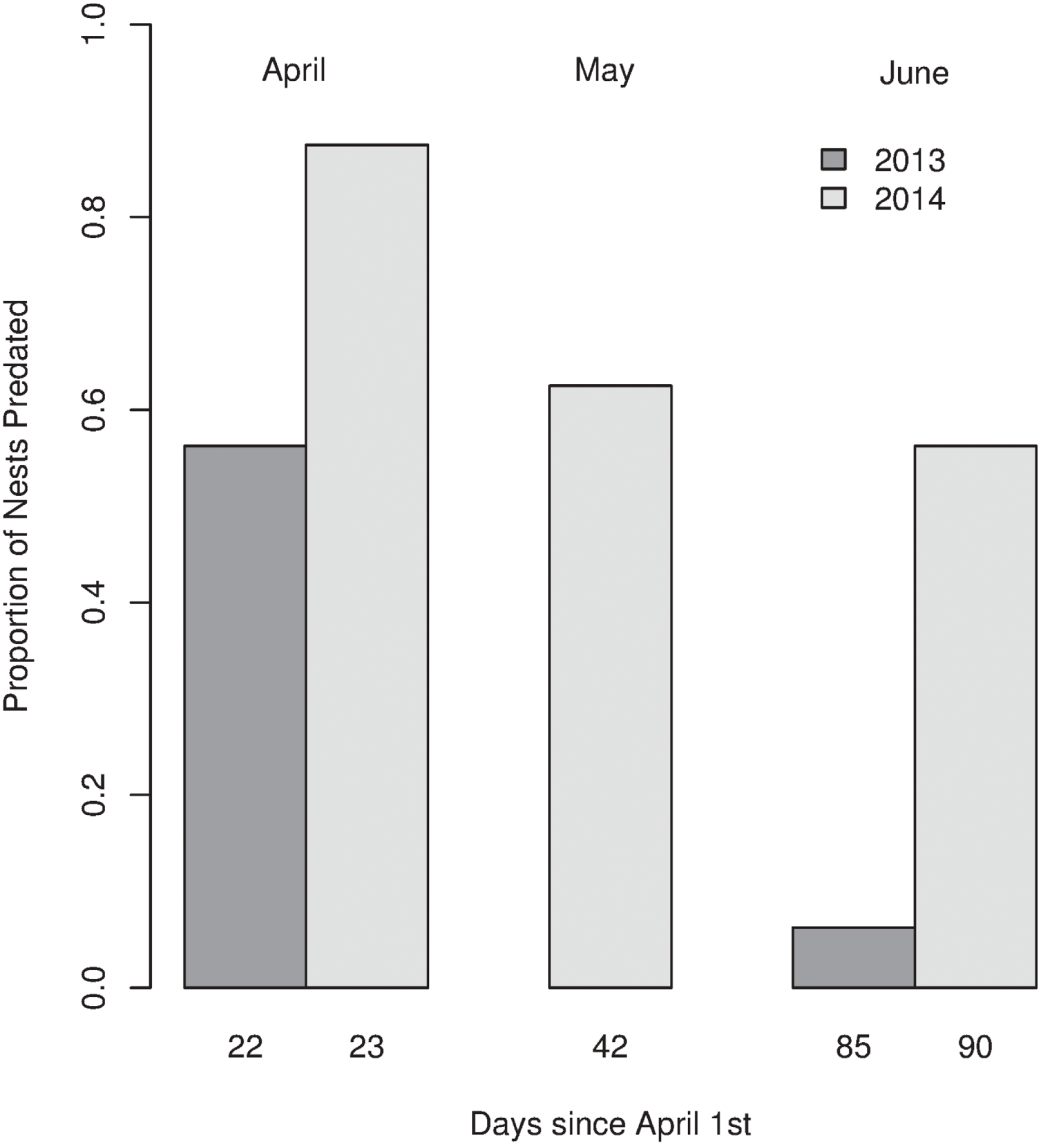
Proportion of nests predated during each experimental repeat and the number of days since April 1st that the repeat was conducted.

**Fig 4:**
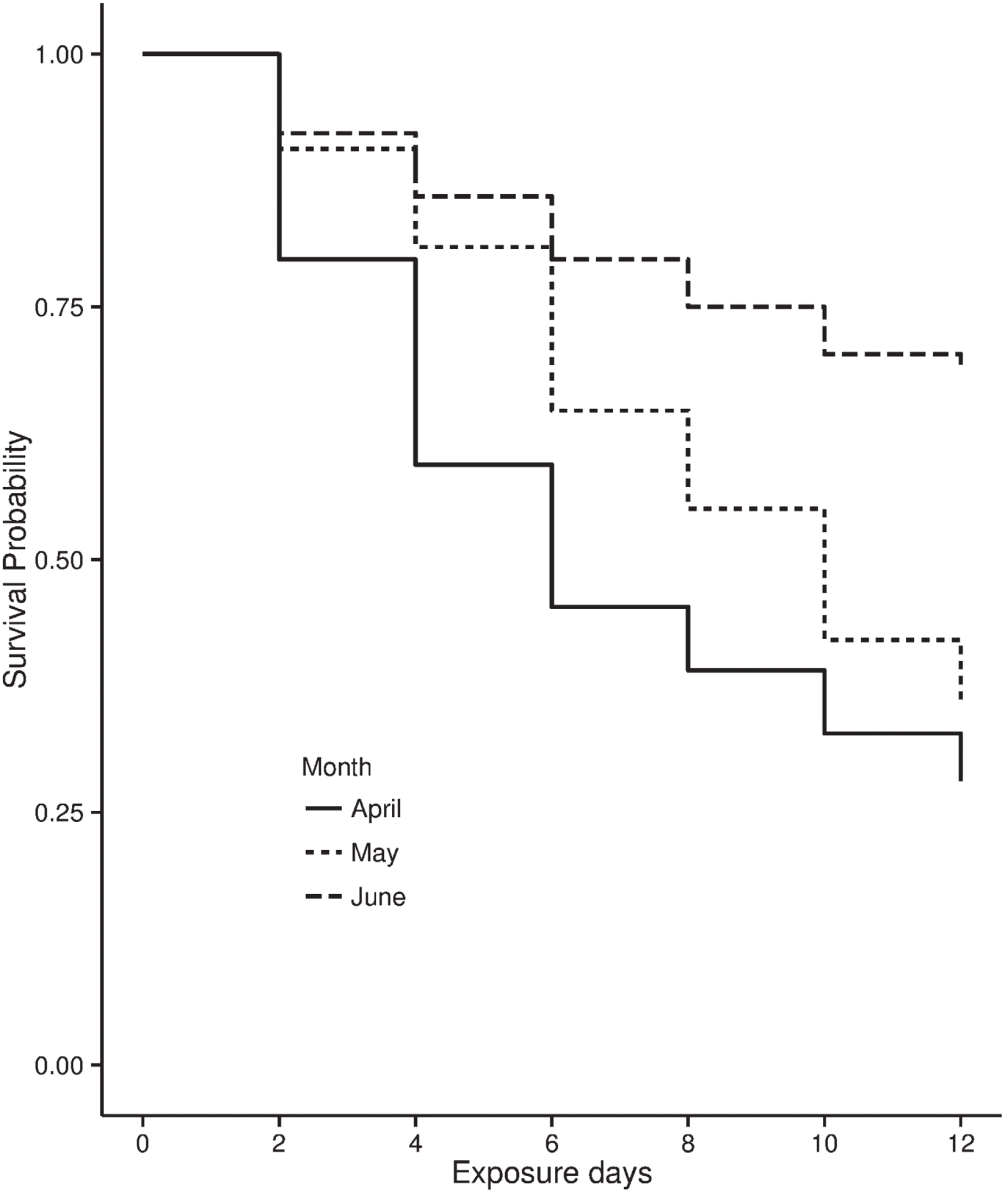
Kaplan-Meier survival functions for nests placed in April, May and Jun.

Overall 53% of the artificial nests placed throughout the study saw a predation event. 2013 saw fewer nests predated (31.2%) than 2014 (68.75%) (Fig 3). When pooling the nests over years, April saw a higher proportion of nests predated (71.8%), than May and June (62.5%, 31.2%, respectively). The Kaplan-Meier survival functions showed a decrease in the proportion of nests predated over the months of the study (*Log-Rank Test; D*.*F = 2*, *χ* ^***2***^ *= 23*.*1*, *P < 0*.*001)* (Fig 4). Table 4 details the hazard ratios and the P-Values of the covariates included in the full Cox proportional hazards model and highlights the most important covariates after the backward stepwise model selection procedure. The most important covariates were the distance of the nest from the edge of the cut area, PC1, PC2 and the year of the study. The only identified predator was a water rail, *Rallus aquaticus*, which was recorded taking a quail’s egg (S2 Fig). There was no evidence of mammalian predation on either of the nest cameras or from teeth or claw marks left upon the clay eggs. Unfortunately, no further information could be collected from the clay eggs, as on predation events they tended to be left intact or removed from the nest completely.

**Table 4:** Hazard ratios, associated 95% CIs, Wald Statistics and P-values of all the effects included in the full model.

Covariate^a^	ΔAIC^b^	Hazard Ratio	95% CI	Wald Statistic	P Value
**PC2**	**0**	**0.57**	**0.47–0.71**	**-5.30**	**< 0.001**
**PC1**	**0**	**0.62**	**0.52–0.74**	**-5.23**	**< 0.001**
**Year**	**0**	**6.4**	**3.64–11.39**	**6.42**	**< 0.001**
**Distance**	**0**	**0.91**	**0.86–0.97**	**-3.13**	**0.0017**
**PC3**	**0**	**0.83**	**0.65–1.05**	**-1.15**	**0.12**
Leaf Litter	1.68	1.00	0.98–1.03	0.57	0.57
Distance:PC1 (Interaction)	1.28	1.00	0.95–1.04	-0.36	0.72
Water Cover	2.00	0.99	0.98–1.01	-0.20	0.84

^**a**^ Terms included in the most supported model are shown in bold.

^**b**^ ΔAIC values are the difference between the AIC of the most supported model and the same model including the effect in question.

## Discussion

We tested three hypotheses with the aim to demonstrate the importance of considering dynamic edge effects in mosaic managed reedbed systems. We first established the presence of an edge effect on nest predation along the edges of cut patches of reed. The most supported CPH models predicted a significant reduction in predation rates with increasing distance from the edge of the cut area. Several other studies have demonstrated similar effects along a variety of natural edges within reedbeds [4,8,25,30]. This edge effect is likely to be driven by a change in species interactions where there is an increase in the rate of encounter between predators and nests along the cut and uncut boundaries.

Such increased encounter rates could occur through several mechanisms. Suvorov et al. [10] suggest that spillover of predators would explain the existence of edge effects in reedbeds if predation rates in neighbouring habitats and at the boundary were higher than in reedbed interior. Alternatively, predation rates that become more dilute towards the interior of both habitats would suggest higher predator activity at boundaries. Since cut reed at low stages of growth does not offer adequate nesting habitat [23,35], we could not compare predation rates in both cut and uncut habitats, but found a clear increase in survival with distance into the reedbed. We suggest that a combination of both spillover mechanisms and boundary attraction could drive edge effects in mosaic cut reedbeds. Neighbouring habitats, such as farmland in this study, can support complex predator communities [69] that may move into the cut areas when accessible. In addition, edges in the study system were non-gradual, with cut reed bordering on old, homogeneous uncut reed, as confirmed by a lack of correlation between habitat variables and distance from the edge. The edges of cut areas will therefore present a dense barrier of reeds to many predatory species, potentially increasing movements parallel to the boundaries [70,71].

We then established whether nest predation rates changed consistently over the season in the two years of the study. The seasonal repeats clearly identified a seasonal decline in artificial nest predation rates (Figs 3 and 4). This seasonality was best modelled through new reed growth (PC1). *Phragmites* reedbeds at temperate latitudes begin new reed growth in April, maturing by June or July [37]. This meant that during the nest experiments undertaken in April, cut squares were accessible with no new reed biomass. By June new growth was dense and around two thirds of its height at full maturity. The changing accessibility of the reedbed through the cut patches could therefore be a major driver of predation rates. Further, Batáry et al. [30] show a reduction in nest predation rates as new reed growth occurs and suggest nests are more difficult to find when new growth is mature. We suggest that new reed growth both reduces the accessibility of the cut areas to predators and makes nests more difficult for predators to locate, discouraging them from searching inefficiently in low payoff areas of dense, mature reed. In April, an increased accessibility will increase the rates of encounter between predators and nests along the boundaries of cut and uncut patches, while later in the season, reduced accessibility and more effectively hidden nests will decrease these encounter rates.

These results clearly stress the importance of considering dynamic processes when investigating nest predation rates and that the fitness implications of habitat use in this system may be time-dependent. Trnka et al. [23], report no differences in predation rates in artificial reed warbler nests between cut and uncut reed stands in Slovakia. However, since comparisons between managed and unmanaged sites can only be made when reed is mature, as early growth does not offer adequate nesting sites, these results cannot consider effects over the full season. By using only the edges of cut areas, we were able to answer Trnka’s [23] call for an investigation into the effects of mosaic reedbed management on nest predation rates throughout the whole breeding season and demonstrate that such effects are temporally dynamic. Species such as the bearded reedling will begin breeding before reed growth has initiated. In several species, recruitment rates of early broods are higher than in later broods [72–75], making them vital for overall breeding productivity. Thus, increased predation rates of early broods in mosaic managed reedbeds could have important fitness implications for birds breeding in this habitat.

With the third hypothesis we aimed to demonstrate a dynamic edge effect in this system by observing the interaction between the effect of the distance from the edge of the cut boundary, and the temporally changing reed structure (PC1). If edge effects are intrinsically related to the accessibility of the reed, and this changes dramatically over the season, we would have expected edge effects to be accentuated in the earlier months of the study when there was no new growth in the cut areas. Despite the strong seasonal effects on predation rates, there was no statistical support for an interaction between distance from the edge and the extent of new growth (PC1). This suggested that any edge effects in this system had a consistent impact in each seasonal repeat and that the dense new growth did not diminish the importance of the boundary between previously managed and unmanaged reed. This edge effect was therefore less dynamic than originally expected, a result that directly emphasises the difficulties in predicting edge effects and the need for a full understanding of the study system when defining an edge effect.

For example, the reason for the unexpected consistency of the edge effect throughout the season in this system could be the continued structural differences between patches of cut and uncut reed even later in the season when reed is mature. The habitat data collected from areas outwith the nests demonstrated that there were considerable changes in the structure of reed between cut patches, and uncut reed when mature. In patches that had been cut reed grew back at a higher density and eventually to a lower height (see also [35]). Therefore, the edges of the cut areas are likely to still be apparent to predators in June when reed is mature. Aerial predators such as the marsh harrier, *Circus aeruginosus*, are important predators in reed systems [4,30,76–78] and could benefit from the inhomogeneity of cut mosaics when hunting. This effect could persist even with the less distinct boundaries at more mature growth. The study also suggested water rail can contribute to nest predation in this system. This species is a reed specialist, reported to prefer predating nests in the interior of the reedbed [77]. In our study, edges created by reed cutting are temporal boundaries within the interior of the reedbed and so water rail could be an important pressure, regardless of the state of reed growth.

Overall, the experiment provided little insight into the main predators of the artificial nests. Despite this, it is important to consider the changing behaviours of predators as potential causes of variation in predation rates. Some predator guilds can show an avoidance of research activity around nests [79,80], which may explain the lack of evidence of mammalian predation in our study. However, as previous studies in reedbeds also show little mammalian nest predation [30,77], and overall nest predation rates were relatively high in this study, we suggest this potential behavioural bias is mitigated. Additionally, seasonal variation in predation rates may be driven by temporal changes in predator behaviour or community. Further identification of the main predators in this system is needed in order to establish the importance of temporal changes in predator behaviours, and how these affect nest predation rates.

While the results show both spatial and temporal dynamics have an important role in reedbed nest predation, we also identified more static aspects of habitat that were associated with nest predation. The reed characteristics represented in PC2 were the density of old reed and the stagger rating for the reed surrounding the nest. These qualities concern only the old, dead reed stems, and are therefore unrelated to both seasonal changes in reed structure and the distance from the edges. They normally occur together when several years of growth accumulate, with old dead stems eventually becoming flatter. Nests in the most dense and staggered reed patches saw lower predation rates. This is probably due to the difficulties in locating nests within the most impenetrable, compacted areas of reed. Generally studies have shown an increase in survival of both real and artificial nests in the most dense areas of reed [12,22,81–84]. However, few have combined this with a rating for reed stagger, which could have implications for aerial predators as these flattened areas obscure nests from above. These results highlight the importance of preserving such reed characteristics during management.

Although not directly investigated, the results also suggested a wider temporal dynamic in nest predation rates. 2014 saw higher predation rates than 2013. This may be explained by changing nest densities. Hoi and Winkler [31] use artificial nests in reedbeds to demonstrate that predation rates increase as the density of nests increases. They suggest that when nest density is high, there is a higher payoff for predators during foraging [12]. While the number of artificial nests placed in each experimental repeat was consistent, bearded reedlings are renowned for fluctuating population sizes [85–87]. Despite similar ringing effort between years, total numbers of new bearded reedlings ringed on the Tay increased by over 150% between 2013 and 2014. Thus, the clear increase in predation rates could have been driven by more predators exploiting the higher density of breeding birds during 2014.

Another possible driver of changes in predation rates between years, may have been differences in the phenology of reed growth. In 2013 a cold start to spring delayed the initiation of reed growth, with new shoots not appearing until late April. In 2014, the reed shoots appeared at that start of April. This is unlikely to have invalidated comparisons of nest predation between years in this study, as the experimental repeats occurred when reed was at a similar growth stage in each year (Fig 2). Similarly, the only habitat variable that significantly changed with the year of the study was leaf litter, which, in further analysis, was seen to have little effect on predation rates. Clearly, further yearly repeats would assist our understanding of the long term changes in predation rates. These would also control for the potential that predation rates were biased during a given experimental repeat by a single predatory individual learning to exploit the artificial nests.

The results contribute to discussion concerning effective reedbed management. Management practices should consider both the benefits and disadvantages of reed cutting. Resistance against reedbed succession may offer long term stability of the reed stand [23,32]. Further, areas of new growth after reed cutting may offer effective foraging opportunities for birds [33,88]. However, studies have shown a reduction in the numbers of many bird species in managed areas [33,89,90], which may be driven by reduced invertebrate abundances [35,36] or delays to breeding [74]. A compromise may be found in the form of mosaic reedbed cutting which may preserve invertebrate abundance and impede succession [23]. We emphasise, however, that the edge effects on nest predation caused by mosaic cutting could have adverse effects for breeding birds, especially those breeding before new reed growth has matured, and that these effects should be considered in future managmenent.

The study shows that several factors contribute to the survival of artificial nests in mosaic cut reedbeds. These are a combination of spatial (edge effects), temporal (seasonal changes in the new reed growth), and static (the structure of the old reed) processes. Despite predictions, spatial edge effects were unaffected by seasonal temporal changes and so the edge effect itself was less dynamic than originally predicted. In natural systems, it is likely that birds have evolved to cope with increased nest predation rates at edges using traditional environmental cues to avoid them. For example, in reedbed systems, natural reed edges have clear gradients in reed density as the reedbed expands outwardly [91]. Animals may be further adapted to cope with dynamic edge effects, if variation is consistent and predictable. Thus, it is essential that for systems subject to anthropogenic influence, where edges may be unpredictable both spatially and temporally, and where traditional environmental cues may be misleading [92], that we understand the implications of edges, and the dynamic processes underlying them.

## Supporting Information

S1 Fig Example of artificial nests used in the study. Left: artificial nest; top right: artificial nest with eggs; bottom right: real bearded reedling nest from the Tay Reedbeds.
(PDF)

S2 Fig Water rail, *Rallus aquaticus*, predating an artificial nest. (PDF)
