# Supplementary material for: The Seasonal Dynamics of Artificial Nest Predation Rates along Edges in a Mosaic Managed Reedbed
Source: PLoS One. 2015 Oct 8;10(10):e0140247. doi: 10.1371/journal.pone.0140247 (PMC4598152; doi:10.1371/journal.pone.0140247)
Supplement: S1 Fig — Left: artificial nest; top right: artificial nest with eggs; bottom right: real bearded reedling nest from the Tay Reedbeds. (PDF) [file pone.0140247.s001.pdf]

Supporting Information 1

Manuscript – **The seasonal dynamics of artificial nest predation rates along edges in a mosaic managed reedbed**

Iain A Malzer[1]*, Barbara Helm[1]

[1]*IBAHCM, University of Glasgow, University Avenue, Glasgow G12 8QQ*

**Email: i.malzer.1@research.gla.ac.uk*

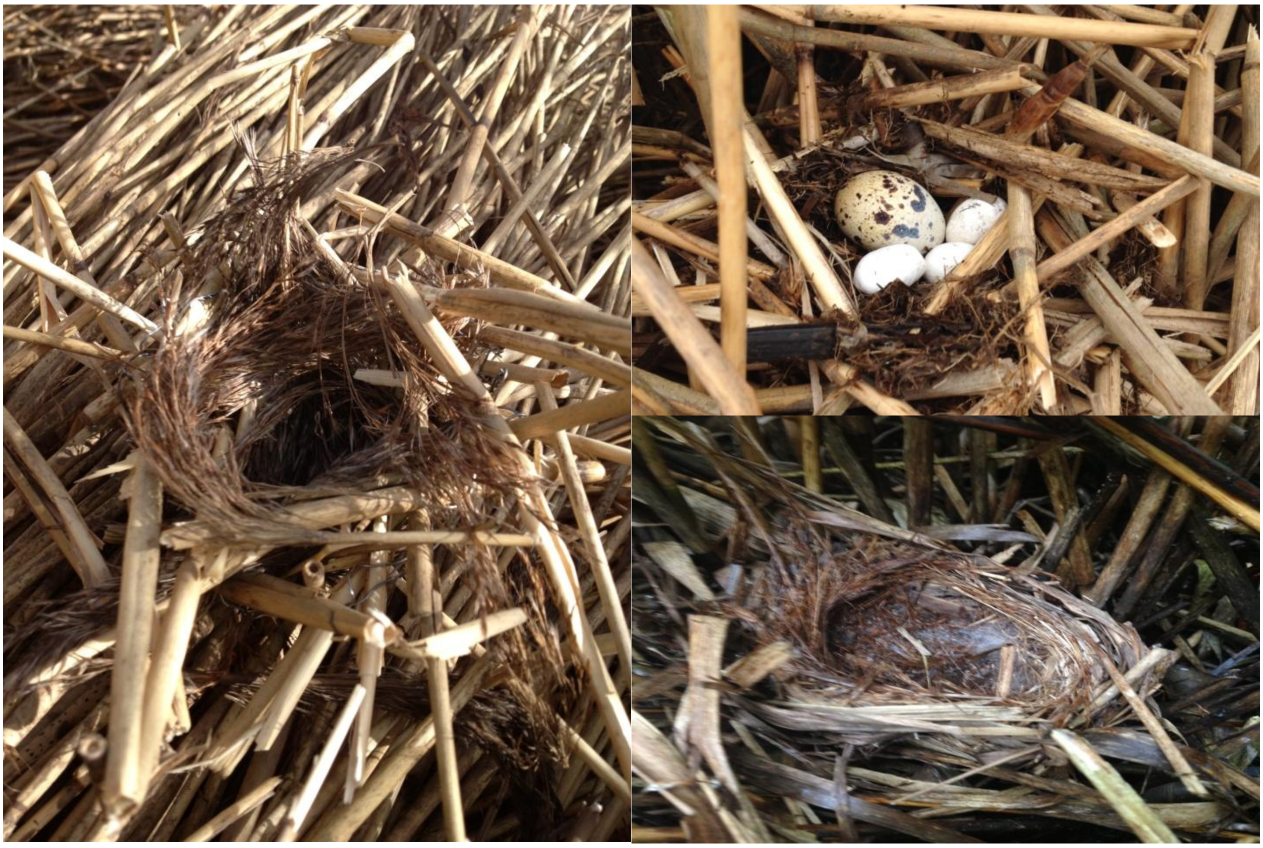

Supporting Information 1 – Left: Artificial nest, Top Right: Artifical nest with eggs, Bottom Right: Real bearded reedling nest found in the Tay Reedbeds.
