# Supplementary material for: The Seasonal Dynamics of Artificial Nest Predation Rates along Edges in a Mosaic Managed Reedbed
Source: PLoS One. 2015 Oct 8;10(10):e0140247. doi: 10.1371/journal.pone.0140247 (PMC4598152; doi:10.1371/journal.pone.0140247)

Supplementary Material 2 - Water *rail, Rallus aquaticus, predating quail's egg in the May 2014 artificial nest experimental.*

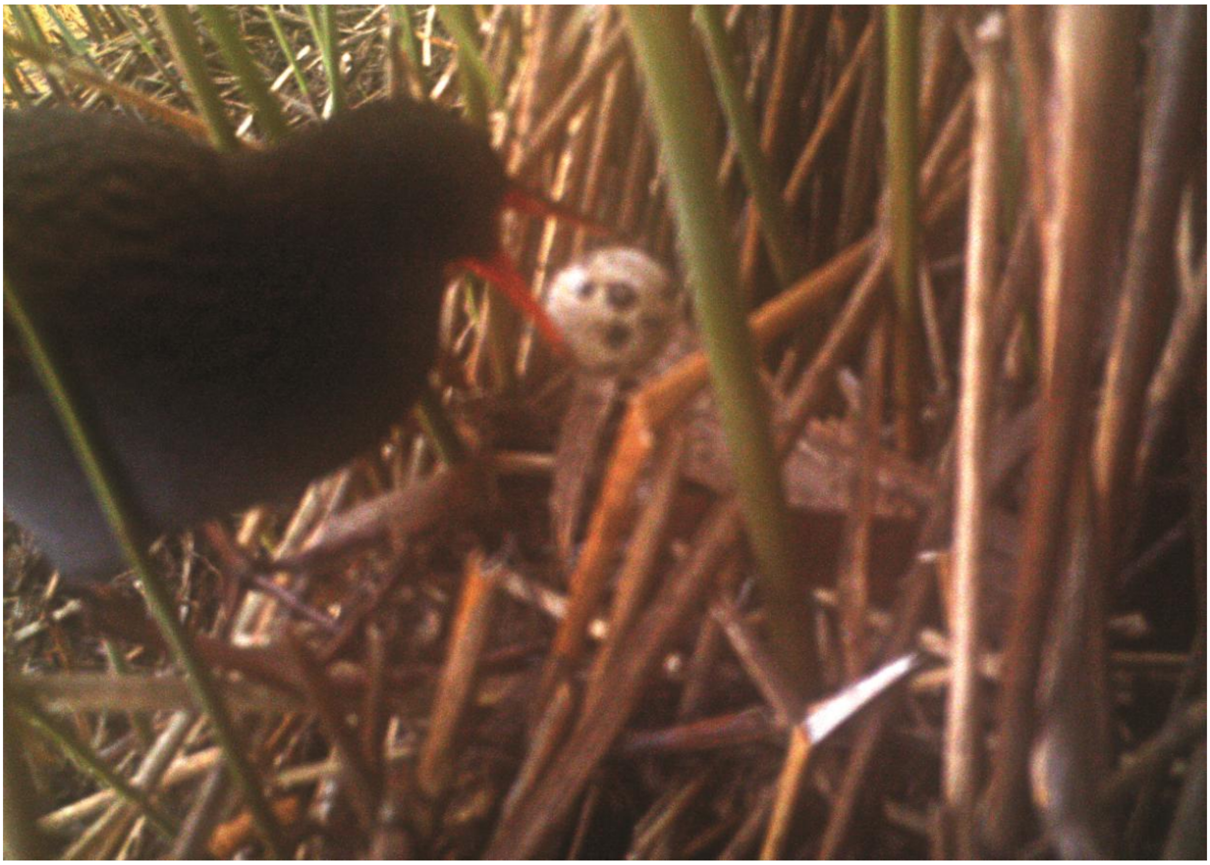

Supplement: S2 Fig — (PDF) [file pone.0140247.s002.pdf]
